# Comprehensive analysis of pre-mRNA alternative splicing regulated by m6A methylation in pig oxidative and glycolytic skeletal muscles

**DOI:** 10.1186/s12864-022-09043-0

**Published:** 2022-12-06

**Authors:** Baohua Tan, Jiekang Zeng, Fanming Meng, Shanshan Wang, Liyao Xiao, Xinming Zhao, Linjun Hong, Enqin Zheng, Zhenfang Wu, Zicong Li, Ting Gu

**Affiliations:** 1 National Engineering Research Center for Breeding Swine Industry, College of Animal Science, South China Agricultural University, 510642 Guangzhou, China; 2 Guangdong Provincial Key Laboratory of Agro-Animal Genomics and Molecular Breeding, College of Animal Science, South China Agricultural University, 510642 Guangzhou, China; 3 State Key Laboratory of Livestock and Poultry Breeding, Guangdong Key Laboratory of Animal Breeding and Nutrition, Institute of Animal Science, Guangdong Academy of Agricultural Sciences, 510640 Guangzhou, Guangdong People’s Republic of China; 4 State Key Laboratory for Conservation and Utilization of Subtropical Agro-bioresources, 510642 Guangzhou, China

**Keywords:** Pigs, Muscle fiber conversion, Alternative splicing, m6A

## Abstract

**Background:**

Different types of skeletal myofibers exhibit distinct physiological and metabolic properties that are associated with meat quality traits in livestock. Alternative splicing (AS) of pre-mRNA can generate multiple transcripts from an individual gene by differential selection of splice sites. N6-methyladenosine (m6A) is the most abundant modification in mRNAs, but its regulation for AS in different muscles remains unknown.

**Results:**

We characterized AS events and m6A methylation pattern in pig oxidative and glycolytic muscles. A tota1 of 1294 differential AS events were identified, and differentially spliced genes were significantly enriched in processes related to different phenotypes between oxidative and glycolytic muscles. We constructed the regulatory network between splicing factors and corresponding differential AS events and identified NOVA1 and KHDRBS2 as key splicing factors. AS event was enriched in m6A-modified genes, and the methylation level was positively correlated with the number of AS events in genes. The dynamic change in m6A enrichment was associated with 115 differentially skipping exon (SE-DAS) events within 92 genes involving in various processes, including muscle contraction and myofibril assembly. We obtained 23.4% SE-DAS events (27/115) regulated by METTL3-meditaed m6A and experimentally validated the aberrant splicing of ZNF280D, PHE4DIP, and NEB. The inhibition of m6A methyltransferase METTL3 could induce the conversion of oxidative fiber to glycolytic fiber in PSCs.

**Conclusion:**

Our study suggested that m6A modification could contribute to significant difference in phenotypes between oxidative and glycolytic muscles by mediating the regulation of AS. These findings would provide novel insights into mechanisms underlying muscle fiber conversion.

**Supplementary Information:**

The online version contains supplementary material available at 10.1186/s12864-022-09043-0.

## Background

Skeletal muscles are composed of different types of fibers that exhibit various heterogeneous characteristics in terms of contractile and metabolic properties. The total number of myofibers remains unchanged after birth. However, these myofibers undergo dynamic processes capable of changing their phenotypes during the growth process [1]. In pigs, muscle fibers are broadly divided into four major types with distinct composition of myosin heavy chain (MyHC) isoforms. MyHC type I and IIa are oxidative fibers, whereas type IIb and IIx fibers are glycolytic and intermediate fibers, respectively [2]. In livestock, the typical fiber-type composition of each muscle is closely related to their postmortem metabolism in the conversion of muscles, thereby influencing meat quality including pH, color, drip loss, tender and fatness [3–5]. The composition ratio of glycolytic fibers in muscles is negatively associated with pH45 value (45 min postmortem), while oxidative muscle fibers are negatively associated with drip loss [3]. Therefore, exploring the mechanism underlying the diverse phenotype of muscle fibers is important to improve meat quality.

RNA methylation N6-methyladenosine (m6A) is the most abundant mRNA internal modification, which is generated by the m6A methyltransferase complex in a highly specific manner that usually occurs within the consensus sequence of RRm6ACH (where R = G or A, and H = A, C or U) [6]. m6A can be recognized by various m6A binding proteins to exert its effects on almost every step of RNA metabolism, including the stability, translation, and splicing of m6A-containing transcripts [6]. It has emerged as a widespread regulatory mechanism that controls gene expression in diverse physiological processes, such as adipogenesis [7] and spermatogenesis [8]. In addition, studies have reported the regulation of m6A in myoblast proliferation and differentiation, and muscle regeneration [9–11]. However, the regulation of m6A in the conversion of skeletal muscle fibers remains largely unknown.

Alternative splicing (AS) is a ubiquitous phenomenon in mammals that leads to the presence of multiple mRNA isoforms or proteins from a single gene. Previous studies reported the high count of differential AS events in skeletal muscle tissues and the dynamic change in AS during myogenesis [12, 13]. The splicing isoforms of several muscle-related genes, such as MSTN, MEF2D, PGC-1α and USP25, exert divergent functions in muscle development [14–17]. Different isoforms within a gene can even act antagonistically to modulate muscle development [14]. AS can also generate gene isoforms with functional diversity in muscle contraction [18–20]. The developmental transitions and muscle-type specific splicing patterns of sarcomere components, including tropomyosin, troponin, and myosin binding protein-C, are well characterized in vertebrate skeletal and cardiac muscles [21, 22]. These results strongly suggest that isoforms switched by AS may be important to fine-tune muscle-type specific properties. AS is a dynamic process and subjected to epigenetic regulation, such as DNA methylation [23], histone modification [24], and non-coding RNA (especially lncRNA) [25]. m6A is also a splicing regulator and significantly affects the AS process by recruiting m6A binding proteins YTHDC1, HNRNPC, and HNRNPA2B1 [26–28]. METTL3-meditated m6A can regulate alternative splicing of gene in spermatogenesis and the development of mammalian cerebellum [29, 30]. YTHDC1 can promote exon inclusion in targeted mRNAs by recruiting splicing factor SRSF3 accompanied with blocking SRSF10 mRNA binding [26]. To date, limited information is known about transcriptome-wide landscape of AS in different types of muscle fibers in pig. Whether m6A can affect diverse biological characteristics between oxidative and glycolytic muscles by mediating the regulation of AS remains unknown.

Our previous study reported m6A methylome profiling of lncRNA in pig extensor digitorum longus (EDL) and soleus (SOL) muscles [31]. In the present study, we further integrated RNA-seq and methylated RNA immunoprecipitation sequencing (MeRIP-seq) for the comprehensive analysis of AS events and m6A methylation pattern. Our study revealed the transcriptome-wide landscape of AS changes, and constructed regulatory network between splicing factors (SFs) and AS events. We highlighted the important role of m6A in muscle fiber conversion by regulating the process of AS. Our study would provide novel insights into exploring biochemical and functional differences between oxidative and glycolytic muscles.

## Results

### Overview of AS events in EDL and SOL

**Fig. 1:**
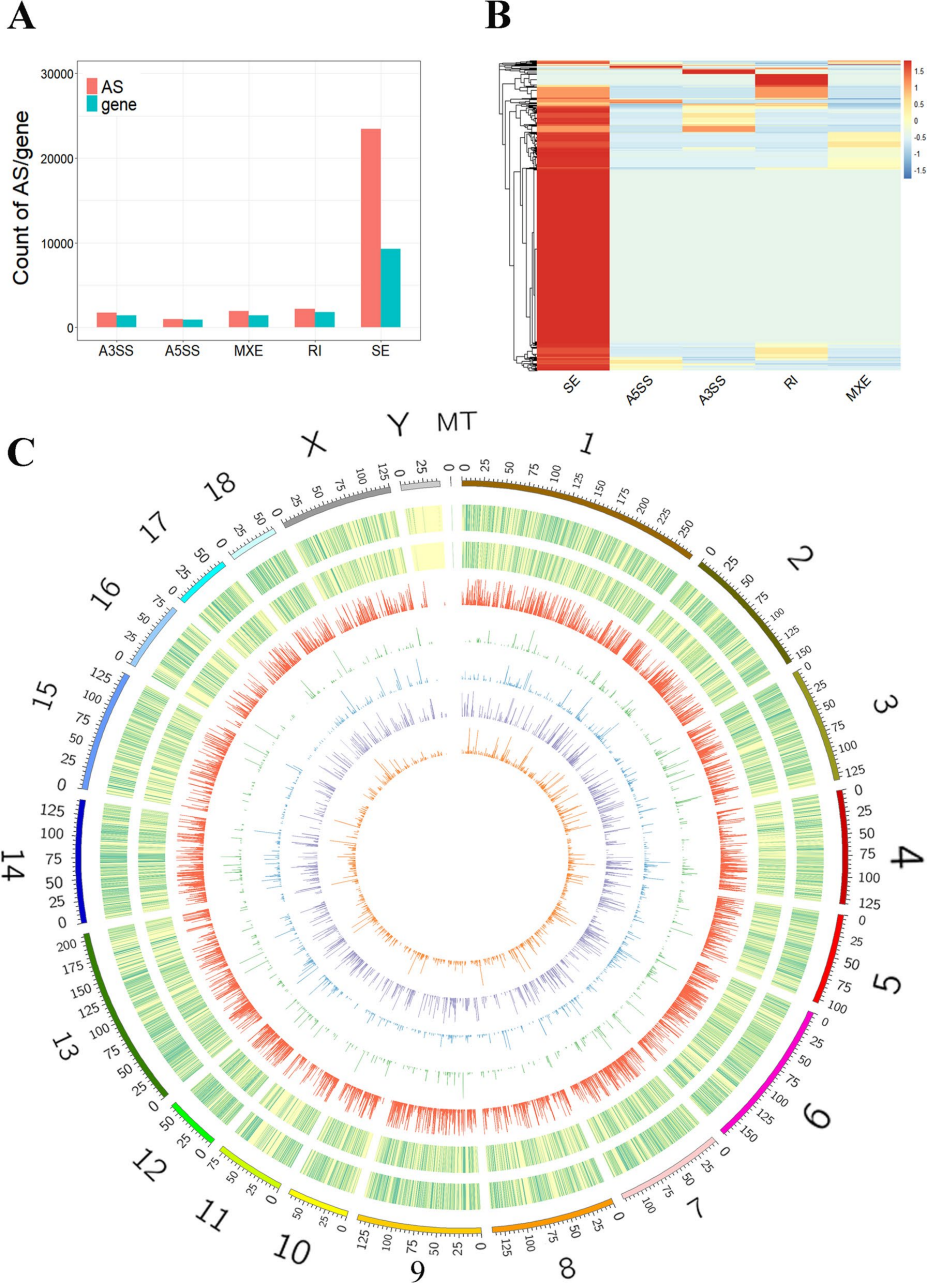
Overview of AS events in the EDL and SOL. **A** Number of different type of AS events and their parent genes. **B** Hierarchical clustering heatmap showing the distribution of different AS events in a single gene. **C** Circos visualization of data corresponding to the chromosomal locations. From outermost ring to innermost ring: (1) Pig chromosomes. (2) Distribution of annotated genes. (3) Distribution of expressed genes. (4) Distribution of SE events. (5) Distribution of A5SS events. (6) Distribution of A3SS events. (7) Distribution of RI events. (8) Distribution of MXE events

Our previous study revealed the difference in phenotypic traits in SOL and EDL and evidenced that SOL and EDL are typical oxidative and glycolytic muscle tissues, respectively [31]. In the present study, the transcriptomes of EDL and SOL muscles were examined to reveal the landscape of AS changes across oxidative and glycolytic muscles. Approximately 464 million reads in total, with an average of 77350202 reads per samples, were obtained from RNA-seq (Additional file 1: Table S1). On average, ~ 96% reads were properly mapped to the pig genome and used for subsequent bioinformatics analysis. The PCA results revealed the clear separation of the replicates in different muscles, and three biological replicates of each muscle were clustered together (Additional file 2: Figure S1). In total, we identified 30353 AS events in 14868 genes, including 23416 SE (skipping exon) events in 9309 genes, 1013 A5SS (alternative 5’ splice site) events in 900 genes, 1778 A3SS (alternative 3’ splice site) events in 1442 genes, 1968 MXE (mutually exclusive exons) events in 1410 genes, and 2178 RI (retained intron) events in 1807 genes (Fig. 1A) (Additional file 3: Table S2). Notably, SE and RI were the most frequently observed AS events, accounting for 84.32% of all AS events, consistent with a previous study on porcine muscles with different intramuscular fat contents [32]. We further explore the distribution of different AS types in a single gene and found that a considerable proportion of genes contained two or more AS events. SE is always accompanied with the presence of A3SS, A5SS, and MXE, while a relatively great proportion of RI uniquely occurs in the genes (Fig. 1B). The distribution of annotated genes, expressed mRNAs, and different types of AS events in the genome is shown in the circos plot, indicating that the AS events were distributed on all chromosomes without obvious chromosome preference (Fig. 1C).

### Landscape and splicing correlation network of differential AS events

**Fig. 2:**
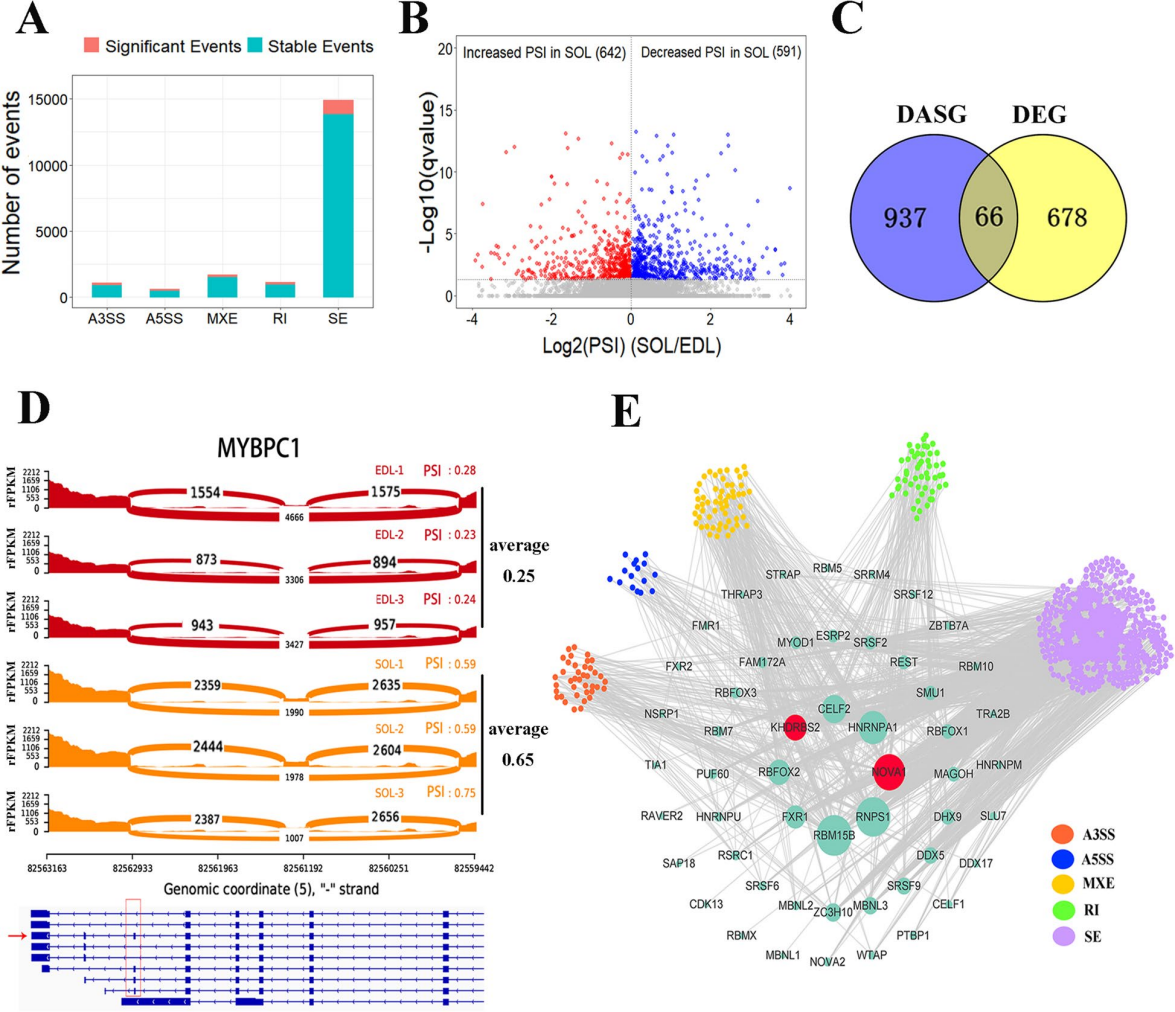
The landscape and splicing correlation network construction of differential AS events. **A** Distribution of different type of DAS events. **B** Volcano plot showing significantly differential AS events. Each point represents one event. PSI: percent-spliced-in. **C** Venn diagram of differentially expressed genes and differentially AS genes. **D** Graphical representation of RNA-seq (sashimi plot) in SOL and EDL in a region containing alternative isoforms. Histogram of PSI level calculated by rMATs is shown in the right of the sashimi plot. A scheme showing the annotated alternative splice isoforms of MYBPC1 is shown in the below of the graphs. Canonical isoform identified in ensembl is labeled with red arrow. Skipping exon is labeled with red box. **E** The high-confidence regulation network between SFs and DAS events. Labeled circles in the center represent SF genes. Red labeled circles indicate differentially expressed SFs. Circles with different colors connected to SF genes by gray lines are distinct types of DAS events

In this study, we obtained 12,514 genes expressed at least in one sample and used for further analysis. Among 1294 differential AS (DAS) events, more than 76% of DAS events are of SE type (Fig. 2A) (Additional file 4: Table S3). The percent‑spliced‑in (PSI) value, representing the fraction of the exon-inclusion variant, was used to estimate the splicing level. There were 642 DAS events with increased PSI level and 591 events with decreased PSI level in SOL compared to EDL (Fig. 2B). A total of 66 intersecting genes between 744 differentially expressed genes (DEGs) and 1003 DAS genes (DASGs) (Fig. 2C) (Additional file 5: Table S4) were identified. Notably, a very small fraction of DASGs was differentially expressed, consistent with the previous reports [33–35]. This suggested that the dynamic AS change in genes has potential to affect muscle heterogeneity in addition to gene dysregulation. MYBPC1 encodes slow myosin binding protein-C, and contributes to the assembly and stabilization of thick filaments [36]. A detailed view of a selected alternative splicing event in MYBPC1 showing RNA-seq read coverage across splice junction is represented in ​Fig. 2D. The inclusion level of the alternative exons in MYBPC1 is decreased from 0.65 to 0.25, corresponding to the reduced expression of functionally canonical MYBPC1 isoform with spliced exons in EDL, although MYBPC1 gene was stably expressed. RNA splicing is regulated by many splicing factors (SFs) to selectively remove introns and join exons [32]. Correlation analysis was performed between SF expression and PSI value of DAS events to investigate the potential regulatory mode between SFs and DAS events and identify key SFs (Additional file 6: Table S5). A total of 641 DAS events corresponding to 639 genes were significantly associated with 52 SFs (Fig. 2E). Several SFs, such as CELF2, RNPS1, KHDRBS2, and NOVA1, were the key nodes in the regulation network and thus recognized as key SFs.

### Functional enrichment of differential AS events

**Fig. 3:**
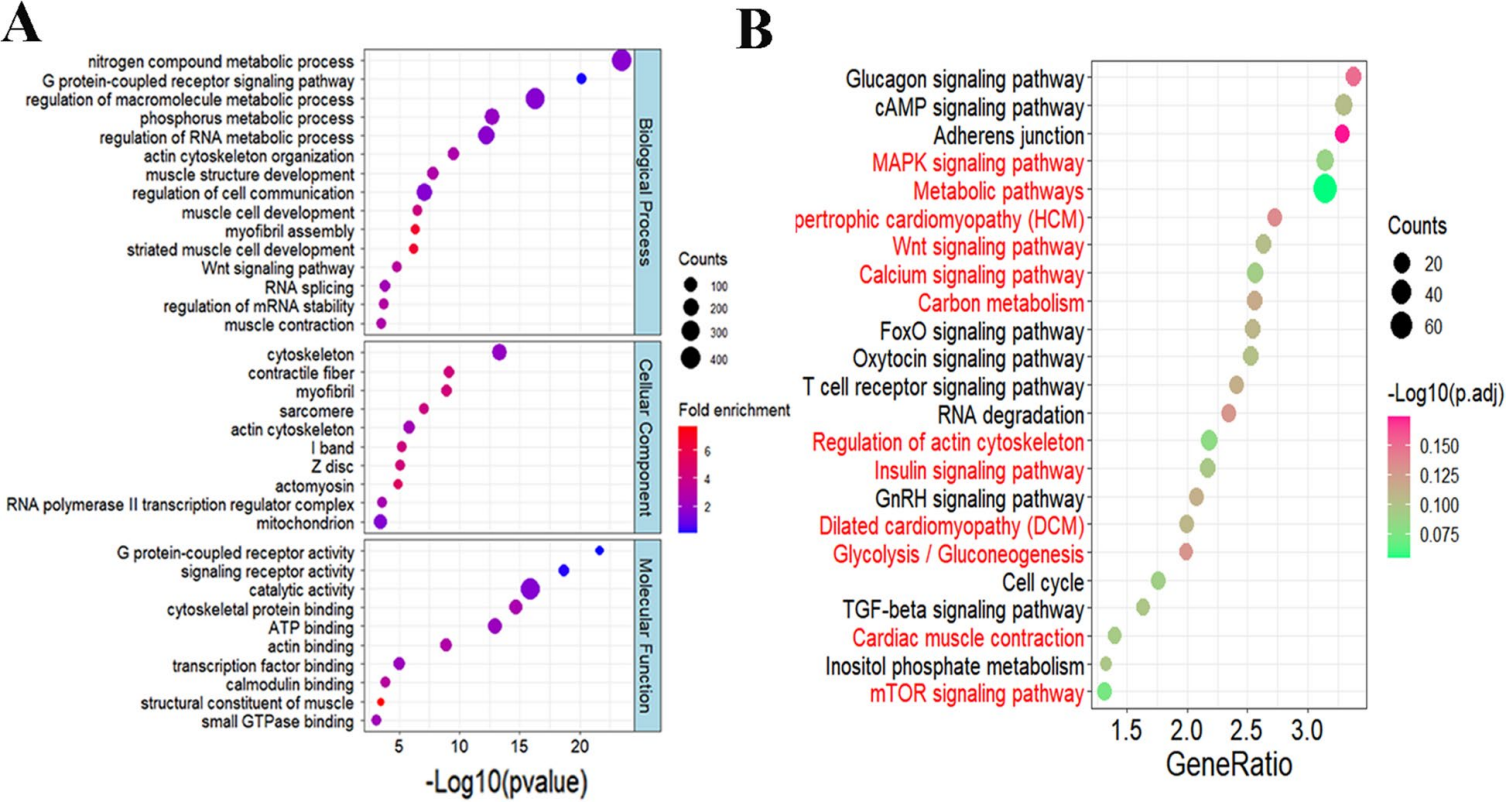
Functional enrichment of differential AS events. **A** The GO enrichment analysis of the SE-DASGs. **B** The KEGG pathway analysis of SE-DASGs

The great proportion of SE in DAS (over 75%) indicated that the dynamic change in SE events may be closely associated with differences between oxidative and glycolytic muscles. Previous studies highlighted the function of m6A in exon skipping [37, 38]. Thus, we further focused on SE events in subsequent analysis. To define the biological functions that are potentially affected by AS in EDL and SOL, we performed GO and KEGG analyses based on the genes with SE-DAS events (Additional file 7: Table S6). GO analysis showed a high enrichment of muscle contractile- and metabolic-related processes affected by AS, including muscle contraction, muscle cell development, and myofibril assembly (Fig. 3A). KEGG pathway analysis revealed the significant enrichment of several metabolic pathways, including MAPK signaling pathway, glycolysis/gluconeogenesis, carbon metabolism, and insulin signaling pathway (Fig. 3B). These results indicated that AS changes in genes may contribute to the different phenotypic traits between oxidative and glycolytic skeletal muscles.

### Regulation of m6A in alternative splicing

**Fig. 4:**
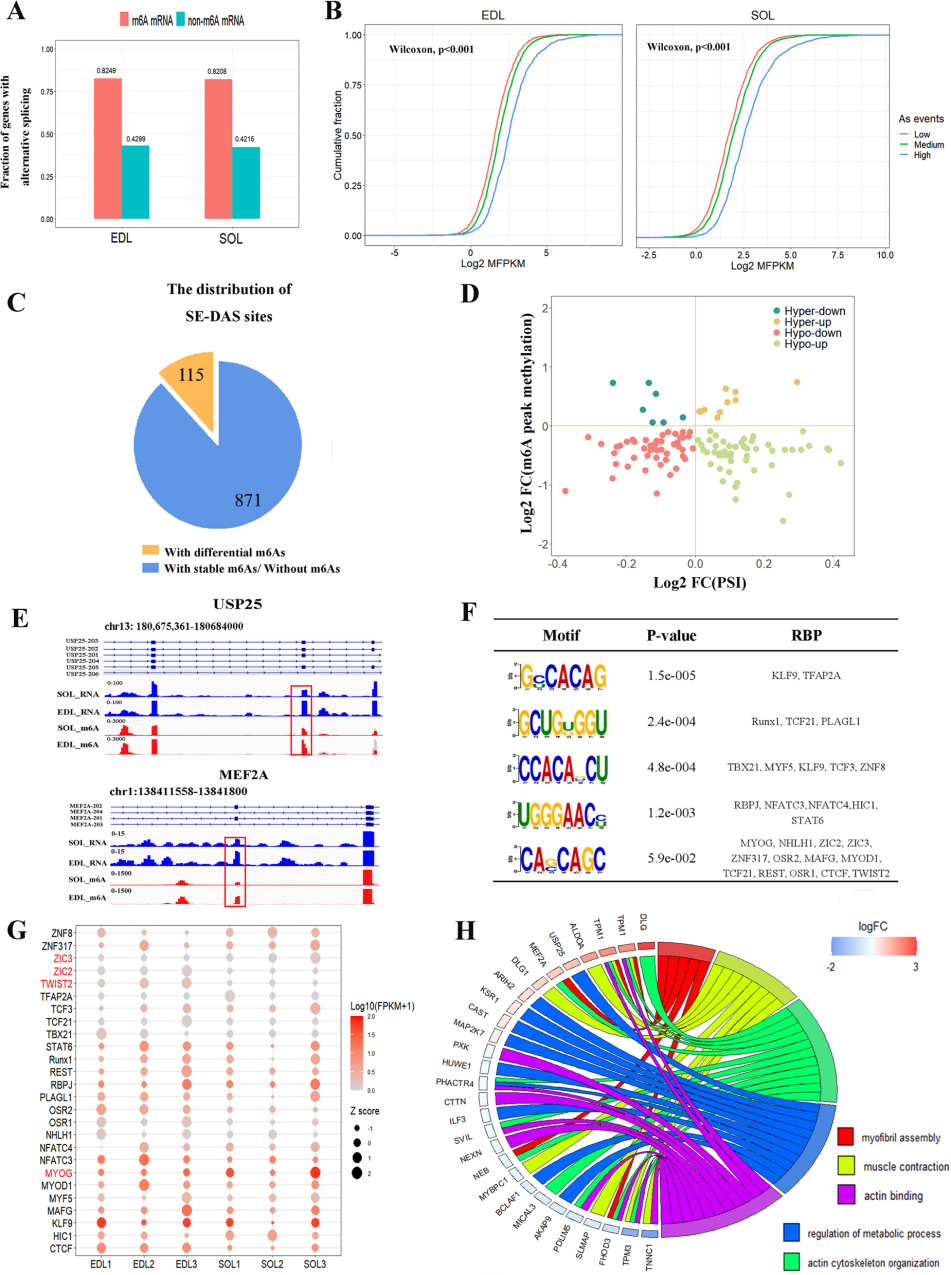
Regulation of m6A in alternative splicing during muscle fiber conversion. **A** The fraction of mRNA with alternative splicing events in EDL and SOL. **B** Cumulative frequency of log2FC (MFPKM) for m6A modified genes with alternative splicing. High (AS Counts ≥ upper quartile; medium (lower quartile < AS Counts < upper quartile); low (AS Counts ≤ lower quartile). **C** The distribution of SE-DAS sites. **D** Four-quadrant graph showing the distribution of skipping exons with a marked change in both PSI and m6A methylation levels in SOL and EDL. **E** Genome browser tracks showing MeRIP-seq (red) and RNA-seq (light-blue) data at gene loci in SOL and EDL. m6A sites was labeled with red box. **F** RBP binding motif enriched in the skipping exon or neighboring regions. **G** Expression heatmap of RBPs. Differentially expressed gene is labeled in red. **H** Chord plot illustrating the GO biological process terms enriched for SE-DMAS genes. Genes contributing to their respective enrichment are shown on the left, and enriched GO terms are shown on the right

To explore the regulation of m6A in alternative splicing during muscle fiber conversion, we performed MeRIP-seq analysis and identified transcriptome-wide m6A sites (hereafter m6As) in SOL and EDL. We obtained 10,128 m6As in 5461 expressed genes, and 11,346 m6As in 5842 expressed genes respectively in SOL and EDL (data not shown). The fraction of m6A-modified genes with AS is about two-fold higher than that of unmodified genes, indicating that m6A-modified mRNA tends to be more likely alternatively spliced than unmodified mRNA (Fig. 4A). Furthermore, we clustered m6A-modified genes into three groups based on the counts of AS events to explore the association between AS events and m6A level. We observed that genes with higher m6A enrichment level had significantly more AS events (Fig. 4B). These findings suggested the crucial regulatory role of m6A in AS during muscle fiber conversion. To further identify which aberrant exon skipping is potentially controlled by m6A, we associated the PSI level with m6A changes in the differentially skipping exon. We finally obtained 115 skipping exons with differential PSI level and m6A enrichment level, corresponding to 92 genes (called SE-DMAS genes) (Fig. 4C) (Additional file 8: Table S7). 55 skipping exons with decreased PSI level were significantly hypermethylated (7) or hypomethylated (48). 60 skipping exons with increased PSI level were significantly hypermethylated (10) or hypomethylated (50) (Fig. 4D). The read coverage of two typical genes, UPS25 and MEF2A, were presented by IGV (Fig. 4E). These data showed that the change in m6A in skipping exon might be associated with distinct exon-skipping level in genes.

We suspected that RNA binding proteins (RBPs) enriched in the neighbor region of skipping exon may participate in the regulation of SE with m6A. We expectedly found a consensus motif RRACH (R: A/ G, H: A/C/U) that is the typical m6A motif conserved in mammals [39–41], indicating the high accuracy in calling m6A peak (Fig. 4F). The binding motif of 26 expressed RBPs was significantly enriched. Some of these factors, such as CTCF, MYOG, and MYOD1, were the well-characterized muscle-specific transcription factors [42] (Fig. 4G). GO functional analysis indicated that SE-DMAS genes were enriched in biological processes related to the conversion of muscle fibers, including myofibril assembly, muscle contraction, and the regulation of metabolic process (Fig. 4H) (Additional file 9: Table S8). These findings suggested that m6A may meditate AS to regulate muscle fiber conversion with muscle-related transcription factors.

### METTL3-mediated m6A regulates alternative splicing of genes functioning in muscle development

**Fig. 5:**
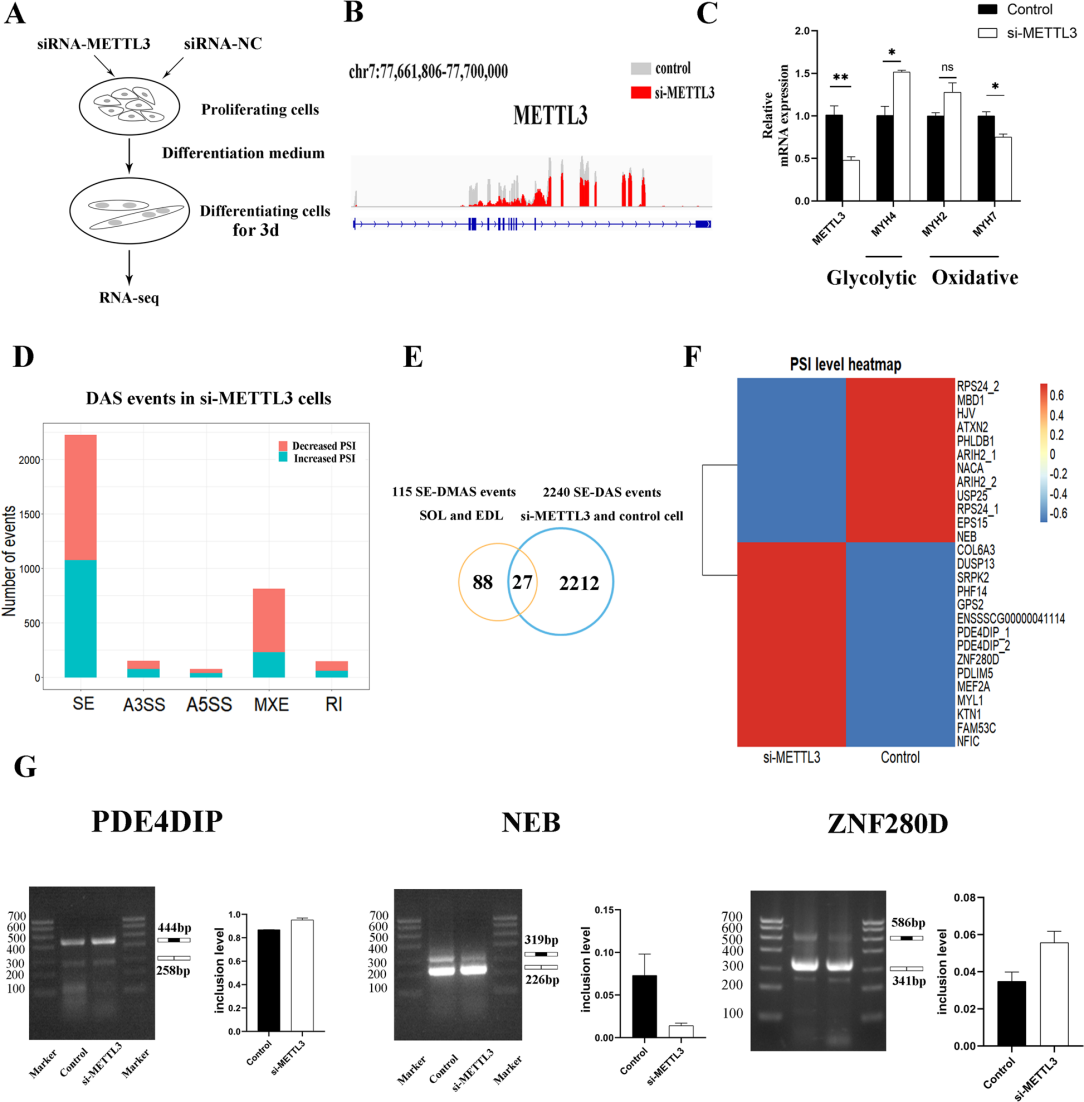
METTL3-mediated m6A regulates alternative splicing of genes functioning in muscle development. **A** A schematic of cell culturing and treatment for sequencing. **B** qPCR results showed that METTL3 expression was significantly reduced in si-METTL3 cells. **C** Genome browser tracks showing RNA-seq read coverage at METTL3 gene body in si-METTL3 cells and control cells. **D** The count of different type of DAS events. **E** Venn diagram of SE-DAS events. **F** The heatmap of PSI level in SE-DAS events. Each row represents one AS events. **G** The exon skipping events were validated by RT-PCR (left panel). PCR primers anneal to exons flanking the skipped exon. Gel electrophoresis images were captured by Tanon-3500 digital gel image system. The inclusion level of SE events was quantified using ImageJ software (right panel), and calculated as: upper band intensity / (upper band intensity + lower band intensity)

We used porcine satellite cells (PSCs) to verify the regulation of m6A in AS. METTL3 is the major catalytic subunit in the RNA m6A methyltransferases complex [43]. We identified the splicing events affected by METTL3 inhibition in PSCs to determine the AS events regulated by m6A (Fig. 5A). A significant downregulation of METTL3 was detected (Fig. 5B-C). The METTL3 knockdown significantly reduced MYH7 gene expression, while increased MYH4 gene expression, indicating the conversion of oxidative fiber to glycolytic fiber. Among the 3144 DAS events, we mainly detected SE events to be enriched, consistent with the previous study in the METTL3 knockdown model (Fig. 5D) (Additional file 10: Table S9) [29]. We intersected these SE-DAS events with the SE-DMAS events identified in SOL and EDL to obtain key AS events regulated by METTL3-mediated m6A (Fig. 5E). A total of 27 SE events within 24 genes were identified, and differential skipping exon events in several spliced genes, including PDE4DIP, ZNF280D and NEB, were validated by RT-PCR (Fig. 5 F-G) (Additional files 11 and 12: Figures S2 and S3). Some of these genes are functioned in muscle development, such as NEB, PDE4DIP, MEF2A, USP25, and HJV [17, 44–47]. In conclusion, our study identified lots of AS events within muscle-related genes that is regulated by METTL3-mediated m6A and provided evidence for the regulation of m6A in AS during the development of muscle fibers.

## Discussion

Animals possess a wide variety of muscle types that support different kinds of movements, from heart beating and digestive peristalsis to fingers flexing [48]. Different muscles exhibit distinct morphologies, metabolic and contractile properties. The question of how muscle diversity is generated during development warrants further explorations. Alternative splicing is a vital post-transcriptional process for precursor RNA to increase the variety of RNA isoforms and proteins. Muscle tissues particularly appear to have high levels of alternative exon use, and splicing patterns notably display muscle-type specificity between cardiac and skeletal muscles [13, 49–51]. This suggests that AS may be important to generate muscle-type specific properties by regulating the inclusion or exclusion of coding sequences in specific genes. Therefore, our study determined the transcriptome-wide analysis of AS change in oxidative and glycolytic muscles. We found that genes with differential AS events were significantly enriched for muscle contraction and metabolism-related processes. These results suggested that AS changes in genes are greatly associated with the conversion of muscle fibers. RNA splicing factors can bind directly to the cis-acting elements of pre-mRNA and regulate their downstream targets in a concentration-dependent manner [52–54]. Thus, a substantial portion of AS events might be subjected to the expression change of splicing factors. We constructed high-confidence regulation network of SFs and DAS events. KHDRBS2 and NOVA1 are dysregulated and potentially regulate a great number of AS events, which might play crucial role in the regulation of AS. Although the relationships warrant to be verified, this network can provide powerful resources for further investigation of AS dysregulation in the conversion of muscle fibers. Collectively, our study presents the association of dysregulated AS events with the phenotype differences between oxidative and glycolytic muscles.

The modification of m6A is the most prevalent and significant RNA modification in mammals and involved in various aspects of biological processes, including RNA splicing. AS can be influenced by the dysregulation of m6A methyltransferases, demethyltransferases and binding proteins [8, 26, 55]. In this study, we built m6A-mediated regulatory networks that control AS to elucidate the function of m6A for AS in various development processes. Our results suggested the importance of m6A in the regulation of AS as a higher proportion of AS occurred in m6A modified genes, which is consistent with the previous study [39]. The change of m6A level may contribute to the differential inclusion level of skipping exon in lots of muscle-related genes, including TNNC1, MEF2A, MYBPC1, USP25, and TPM1. TNNC1 is one of the three slow skeletal muscle troponins, and regulates muscle contraction by mediating the response of muscles to calcium ions [56]. MEF2A is essential for regulating myogenic differentiation and inducing slow skeletal muscle fiber gene expression [47, 57, 58]. Some of these genes have been revealed the distinct roles of different spliced isoforms in muscle development. Ubiquitin-specific protease (USP25) can encode three protein isoforms produced by alternative splicing in human. Only the longer USP25 isoform (USP25m) is specifically expressed in muscle tissues and interacts with three sarcomeric proteins to regulate myogenesis [17]. In this study, USP25 gene was stably expressed in different muscles but occurred differential skipping in exon 19. Consistent with the decreased inclusion of exon 19 in SOL, the proportion of spliced USP25 isoforms with skipping exon 19 is lower in SOL than EDL. In conclusion, our results suggested that m6A may regulate muscle fiber conversion by mediating AS to modulate the relative proportion of spliced isoforms in a gene without expression change.

Many types of RBPs can regulate alternative splicing. Our study found that the binding motif of RBPs, including CTCF, MYOD1, MYOG, and MRF5, were enriched in m6A-modified skipping exon. Myogenic regulatory factor MYOD can convert various differentiated cell types in myogenesis, which is indispensable for muscle-specific alternative splicing in the pre-mRNA of mouse mitochondrial ATP synthase gamma-subunit [59, 60]. Our study unexpectedly revealed the potential role of other myogenic regulatory factor, MYOG and MRF5, in the regulation of AS. The chromatin insulator CTCF has been defined as an important regulator of alternative splicing, and its binding is correlated with exon inclusion in spliced mRNA [61]. The enrichment of CTCF binding sites proximal to alternatively spliced exons is affected by DNA methylation, RNAPII elongation, histone modification and splicing factor recruitment [62]. Our study revealed the significant enrichment of m6A in CTCF binding sites within skipping exon, suggesting the potential co-regulation of CTCF and m6A in the AS process. Although the cooperative/competitive or dependent/independent relationships between these factors and m6A-mediated AS needs further experimental validation, the regulatory mechanism between m6A and muscle-related transcript factors in AS is a novel insight to clarify AS change in different muscles.

Satellite cells are a heterogeneous population of muscle progenitors with stem cell properties responsible for the regeneration of adult skeletal muscle. Satellite cells can self-renew, and eventually differentiate through fusion with each other or to distinct myofibers. Different type of myofiber occur conversion as the differentiation goes on [63]. Thus, satellite cell is widely used as ideal model to study muscle fiber conversion [31, 64, 65]. In this study, we down-regulated METTL3, the major m6A methyltransferases, to study the function of m6A in muscle fiber conversion. Our results evidenced the conversion of oxidative to glycolytic fiber after METTL3 inhibition. Betaine that can enhance global m6A level as methyl donor, induces glycolytic to oxidative fiber in vivo and in vitro [66, 67]. These results evidenced that m6A can regulate muscle fiber conversion. In addition, our study verified the differential AS events of several muscle-related genes in different type of skeletal muscles, and the regulation of METTL3-medicated m6A in these AS events. These findings associated the m6A regulation with AS process in muscle fiber conversion, which would provide novel insights into mechanisms underlying muscle fiber conversion.

## Conclusion

Our study provided the transcriptome-wide landscape of AS change and the m6A methylome in different muscles. We associated the regulation of m6A in AS to the differences in the phenotypes between oxidative and glycolytic muscles. The loss of m6A methyltransferase METTL3 could promote the conversion of oxidative to glycolytic fiber. We further validated several AS events in genes involving in muscle fiber conversion that are regulated by METTL3-mediated m6A. These results could provide novel clues to clarify how functionally distinct muscle fiber-types arise and convert during development.

## Methods

### Sample collection

Three 6-month-old male Duroc pigs raised in the same cage were randomly selected from the breeding pig farm of Guangdong Wen’s Foodstuffs Group Co., Ltd. (Yunfu, China). All pigs were fed standard diet three times each day and provided with sufficient drinking water. On November 11, 2021, muscle samples were collected from the intermediate section of the extensor digitorum longus and soleus after pigs were slaughtered under anesthesia. Collected tissues were rapidly sectioned in 2-ml centrifuge tube and frozen in liquid nitrogen.

### Cell culture

For proliferation, porcine skeletal muscle satellite cells (PSCs) were cultured in proliferating medium with RPMI-1640 (Gibco, USA), 20% FBS (Gibco, USA), 1% non-essential amino acids (Gibco, USA), 0.5% chicken embryo extract (GEMINI, USA), 1% GlutaMax (Gibco, USA), 1% antibiotic–antimycotic (Gibco, USA), and 2.5 ng/ml bFGF (Gibco, USA) under moist air with 5% CO_2_ at 37 °C. For differentiation, the proliferating medium was replaced with RPMI 1640 containing 2% horse serum (Gibco, USA) when the cells reach 80–90% confluence.

### RNA oligonucleotides, and cell transfection

Small interfering RNA (siRNA) against pig METTL3 were designed and synthesized by GenePharma (Shanghai, China), and a nonspecific duplex was used as negative control (NC). siRNA oligos sequences were as follows: METTL3 (sense 5’-CCGGUUCAAGCAAAGGUAUTT-3’), NC (sense 5’-UUCUCCGAACGUGUCACGUTT-3’). The transfection was performed with Lipofectamine 3000 reagent (Invitrogen, Carlsbad, CA, USA) according to the manufacturer’s protocol.

### Reverse-transcription (RT) PCR and quantitative RT-PCR

Total RNA was isolated from tissues or cells using TRIZOL reagent (Gibco, USA) and reverse-transcribed to create cDNA by Evo M-MLV RT Kit (AG, China) following the manufacturer’s manual. RT-PCR products were visualized on 2% agarose gels electrophoresis. qPCR was performed on an ABI Quant Studio 6 Flex system (Thermo Fisher Scientific, Waltham, MA, USA). Each reaction mixture (10 µl) contained 1 µl cDNA solution, 0.5 µl of forward/reverse primer, 5 µl SYBR Select Master Mix (Thermo Fisher Scientific, Waltham, USA), and 3 µl ddH2O. Reaction conditions were as follows: 2 min at 50 °C, 10 min at 95 °C, then 40 cycles of 15 s at 95 °C, 10 s at 60 °C, and 15 s at 72 °C, followed by melting curve analysis from 60 to 95 °C to evaluate the specificity of the PCR products. The Ct ^(2−∆∆Ct)^ method was used to analyze relative RNA expression. All primer used in this study was shown in Additional file 13: Table S10.

### MeRIP-seq and RNA-seq library preparation

MeRIP-Seq was performed in accordance with our previous study [68]. In briefly, RNA fragments were incubated for 2 h at 4℃ with m6A-specific antibody (Synaptic Systems, Germany) in IP buffer (50 mM Tris-HCl, 750 mM NaCl and 0.5% Igepal CA-630). A portion of the initial fragmented RNA was performed to RNA-seq and used as the input library for MeRIP-seq. The m6A-Ab mixture was incubated with protein magnetic beads (Thermo Fisher, USA) at 4 °C for 2 h for immunoprecipitation. After washing and elution, m6A IP RNA is obtained. Both the m6A IP RNA and the input RNA were used for library generation with NEBNext® Ultra™ Directional RNA Library Prep Kit (New England Biolabs Inc., USA). The average insert size for the final cDNA library was 300 ± 50 bp. At last, we performed the 2 × 150 bp paired-end sequencing (PE150) on the Illumina Novaseq™ 6000 (LC-Biotechnology CO., Ltd., China) following the vendor’s recommended protocol.

### RNA-seq analysis

The clean data were produced by removing reads containing adapters, reads containing over 10% of poly (N) by fastq (version 0.23.1) from the raw data [69]. Hisat2 (version 2.2.1) was used to build genome index and mapped clean reads to reference genome of pig (Ensembl Sscrofa 11.1.94) with chain-specific parameters: “rna-strandness RF” [70]. The gene or transcript read counts were calculated by featureCount (version 2.0.1) [71] and normalized to the fragments per kilo-base of exon per million fragments (FPKM) using custom script. Coding genes with ≥ 0.5 FPKM in at least one library were considered as expressed genes and used for further analysis. Differential gene expression analysis was identified using the R package DESeq2 with the criteria log2 fold-change ≥|1| and FDR < 0.05 [72]. Gene ontology (GO) analyses was performed using PANTHER [73]. Kyoto Encyclopedia of Genes and Genomes (KEGG) pathway analysis was carried out in KOBAS-i [74, 75]. Circos plots were generated to visualize the detail of AS events and genes in chromosomes by Circos software (version 0.67) [76]. Identification and quantification of AS events or DAS events were conducted by using the software rMATS (version 4.1.2) with updated GTF files [77]. The visualization of rMATS output is performed in rmats2sashimiplot, and the read density in the plot is represents as rFPKM (https://github.com/Xinglab/rmats2sashimiplot). The DAS events were characterized with a false discovery rate (FDR) < 0.05. rMATS was used to report the percent-spliced-in (PSI) value that represents the inclusion level of the skipping exon.

### MeRIP-seq analysis

The quality control of raw reads and mapping of clean read were performed as RNA-seq analysis. The m6A peaks were identified by the R package exomePeak2 under default parameters settings [78]. Differential methylated peaks were also called via exomePeak2 according to the criteria adjusted p-value < 0.05. Identified peaks were annotated by using bedtools (version 2.29.2) suite and custom shell script [79]. The DREME tool in the MEME suite was used to discover relatively short (up to 8 bp) motifs against the upstream 1 kb to downstream 1 kb of skipping exon [80]. To associate the enriched motifs to potential RBPs, all enriched motifs were compared against a database of known motifs using Tomtom [81]. Read coverage was visualized for select regions in the Integrative Genomics Viewer [82]. R package ggplot2 was contributed to the graphical representation [83]. The m6A enrichment levels of genes were represented as MFPKM (MFPKM = FPKM_IP/FPKM_INPUT) averaged in the three biological replicates.

### Construction of the splicing correlation network

SFs that participated in the process of alternative RNA splicing (GO: 0000380) were obtained from the Molecular Signatures Database (MSigDB) [84]. Spearman’s correlation analysis was conducted to explore the correlations between the expression of the SFs and the PSI level of the DAS events. The P-value was adjusted by the Benjamin–Hochberg (BH) method, and a correlation coefficient ≥ 0.9 and an adjusted P-value < 0.05 were considered statistically significant. The regulatory network between SFs and AS events is generated using Cytoscape (version 3.4.0) [85]. 

## Supplementary Information

**Additional file 1:** **Table S1.** Summary of the mapping of sequencing data.

**Additional file 2:** **Figure S1.** Principal component analysis (PCA) of RNA-seq data on EDL and SOL. EDL muscle samples were within blue circle, and SOL muscle samples were within green circle.

**Additional file 3:** **Table S2.** AS events identified in SOL and EDL.

**Additional file 4:** **Table S3.** Differentially AS events identified in SOL and EDL.

**Additional file 5:** **Table S4.** Differentially expressed genes identified in SOL and EDL.

**Additional file 6:** **Table S5.** Profiling of regulatory splicing correlation network in SOL and EDL.

**Additional file 7:** **Table S6.** GO and KEGG enrichment analysis of the Differentially AS genes.

**Additional file 8:** **Table S7.** The identification and annotation of SE-DMAS sites in SOL and EDL.

**Additional file 9:** **Table S8.** GO enrichment analysis of the SE-DMAS genes.

**Additional file 10:** **Table S9.** Differentially AS events identified in si-METTL3 cells.

**Additional file 11:** **Figure S2.** The exon skipping events of genes in tissues were validated by RT-PCR (left panel). The inclusion level of SE events was quantified using ImageJ software (right panel).

**Additional file 12:** **Figure S3.** Original files of gel images. Original files of gel images. (A) The validation of DAS events in the si-METTL3 and control cells. The first lane to fourth lane is PDE4DIP gene. The sixth lane to ninth lane is NEB gene. (B) The validation of DAS events in si-METTL3 and control cells. The first lane to fourth lane is ZNF280D gene. (C) The validation of DAS events in SOL and EDL. The first lane to fourth lane is PDE4DIP gene. The fifth lane to eighth lane is NEB gene. The ninth lane to twelfth lane is ZNF280D gene. (D) The validation of DAS events in SOL and EDL. The first lane to fourth lane is PDE4DIP gene.

**Additional file 13:** **Table S10.** All primer used in this study.

## Data Availability

The datasets presented in this study are deposited in the NCBI Sequence Read Archive (SRA), the records can be accessed by accession numbers PRJNA810786.

## Abbreviations

m6A: N6-methyladenosine
AS: Alternative splicing
DAS: Differential alternative splicing
SE: Skipping exon
IR: Intron retention
A3SS: Alternative 3´ slice site
A5SS: Alternative 5´ slice site
MXE: Mutually exclusive exon
DEG: Differentially expressed gene
DASG: Differential AS gene
GO: Gene ontology
KEGG: Kyoto Encyclopedia of Genes and Genomes
PSI: percent-spliced-in
FPKM: fragments per kilo-base of exon per million fragments
RBP: RNA binding proteins.
