# Supplementary material for: Comprehensive analysis of pre-mRNA alternative splicing regulated by m6A methylation in pig oxidative and glycolytic skeletal muscles
Source: BMC Genomics. 2022 Dec 6;23:804. doi: 10.1186/s12864-022-09043-0 (PMC9724443; doi:10.1186/s12864-022-09043-0)
Supplement: Supplementary file 2 — Additional file 2: Figure S1. Principal component analysis (PCA) of RNA-seq data on EDL and SOL. EDL muscle samples were within blue circle, and SOL muscle samples were within green circle. [file 12864_2022_9043_MOESM2_ESM.docx]

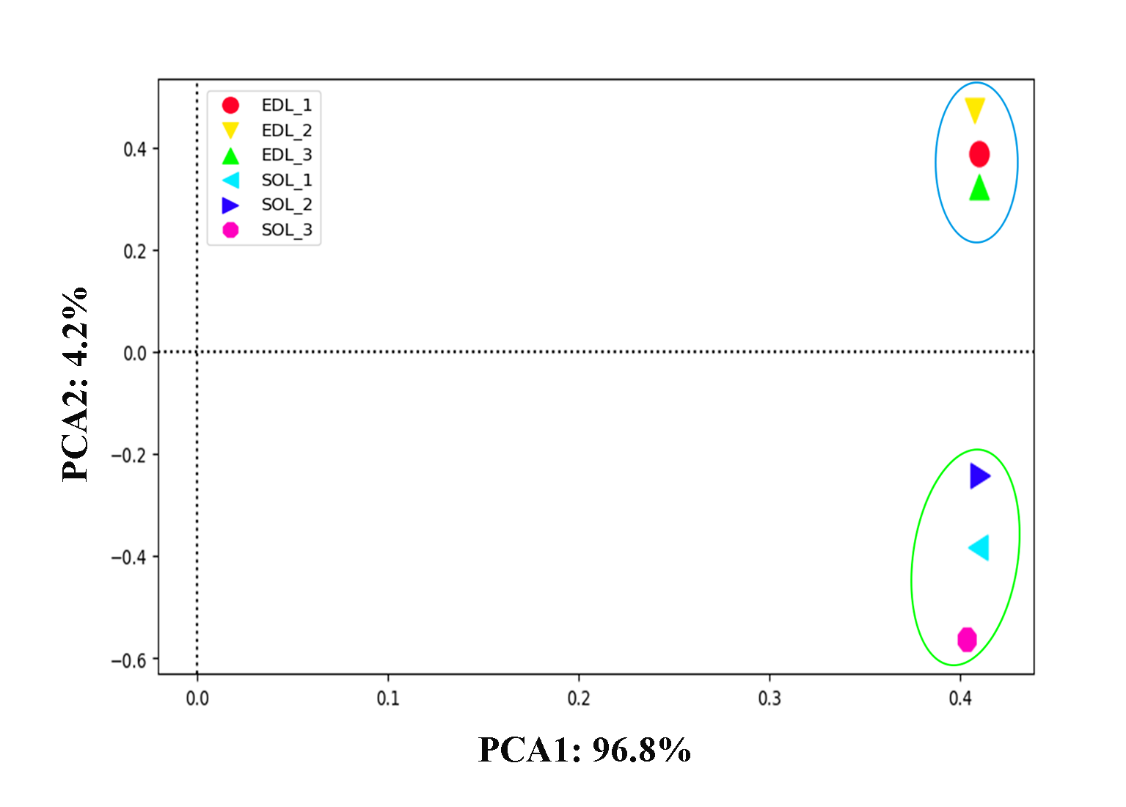


**Additional file 2:** **Figure S1.** Principal component analysis (PCA) of RNA-seq data on EDL and SOL. EDL muscle samples were within blue circle, and SOL muscle samples were within green circle.
