# Supplementary material for: Comprehensive analysis of pre-mRNA alternative splicing regulated by m6A methylation in pig oxidative and glycolytic skeletal muscles
Source: BMC Genomics. 2022 Dec 6;23:804. doi: 10.1186/s12864-022-09043-0 (PMC9724443; doi:10.1186/s12864-022-09043-0)
Supplement: Supplementary file 11 — Additional file 11: Figure S2. The exon skipping events of genes in tissues were validated by RT-PCR (left panel). The inclusion level of SE events was quantified using ImageJ software (right panel). [file 12864_2022_9043_MOESM11_ESM.docx]

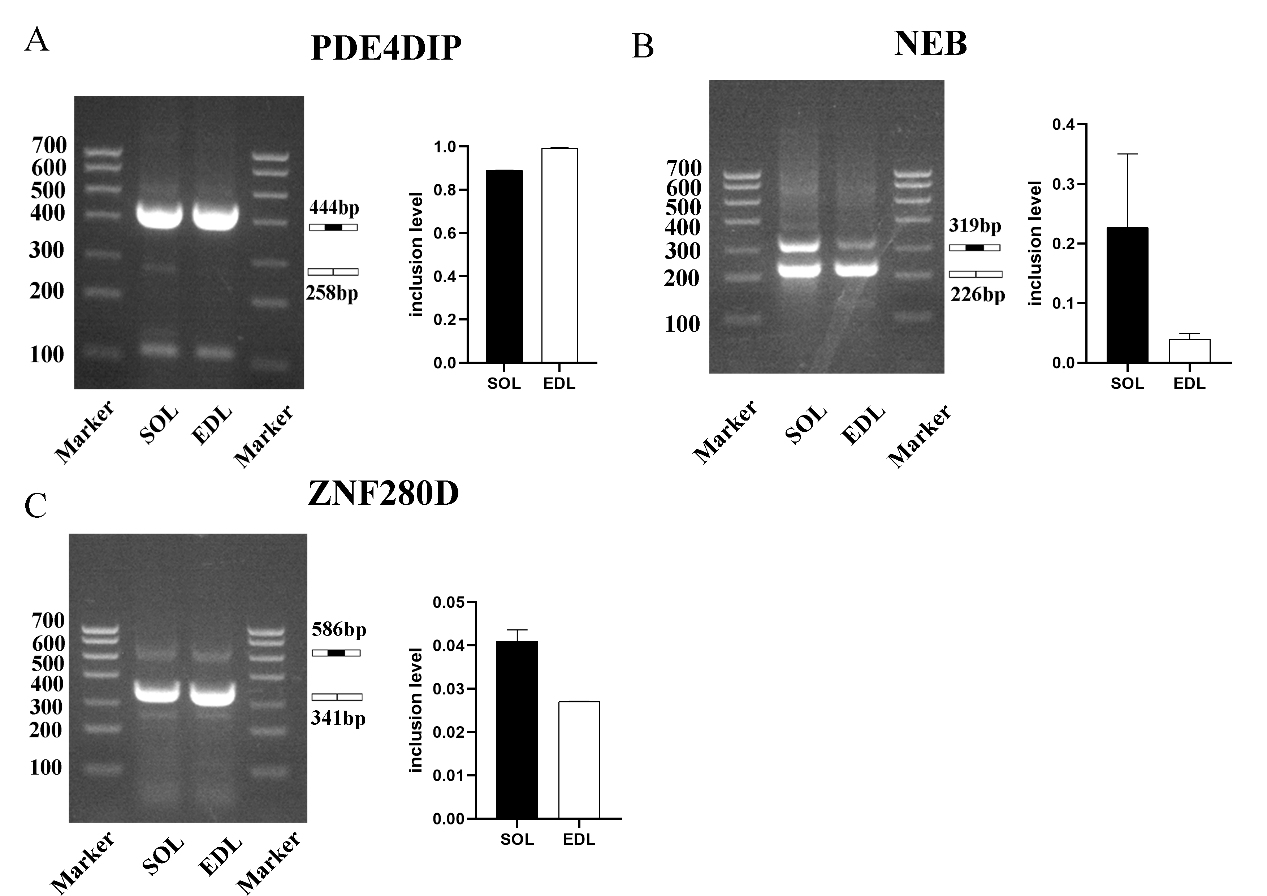


**Additional file 11: Figure S2：**The exon skipping events of genes in tissues were validated by RT-PCR (left panel). The inclusion level of SE events was quantified using ImageJ software (right panel).
