# Supplementary material for: Comprehensive analysis of pre-mRNA alternative splicing regulated by m6A methylation in pig oxidative and glycolytic skeletal muscles
Source: BMC Genomics. 2022 Dec 6;23:804. doi: 10.1186/s12864-022-09043-0 (PMC9724443; doi:10.1186/s12864-022-09043-0)
Supplement: Supplementary file 12 — Additional file 12: Figure S3. Original files of gel images. Original files of gel images. (A) The validation of DAS events in the si-METTL3 and control cells. The first lane to fourth lane is PDE4DIP gene. The sixth lane to ninth lane is NEB gene. (B) The validation of DAS events in si-METTL3 and control cells. The first lane to fourth lane is ZNF280D gene. (C) The validation of DAS events in SOL and EDL. The first lane to fourth lane is PDE4DIP gene. The fifth lane to eighth lane is NEB gene. The ninth lane to twelfth lane is ZNF280D gene. (D) The validation of DAS events in SOL and EDL. The first lane to fourth lane is PDE4DIP gene. [file 12864_2022_9043_MOESM12_ESM.docx]

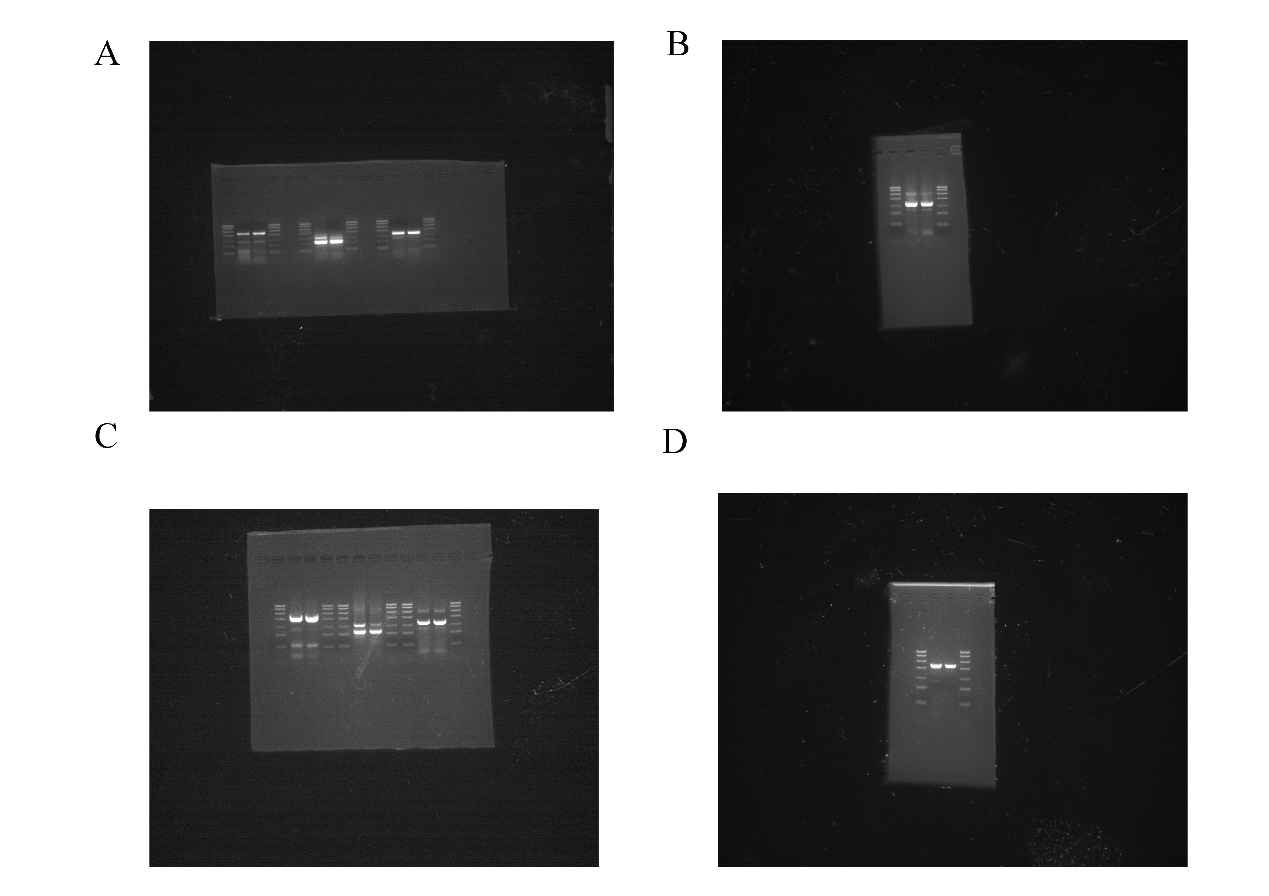


**Additional file 12: Figure S3.** Original files of gel images. Original files of gel images. (A) The validation of DAS events in the si-METTL3 and control cells. The first lane to fourth lane is PDE4DIP gene. The sixth lane to ninth lane is NEB gene. (B) The validation of DAS events in si-METTL3 and control cells. The first lane to fourth lane is ZNF280D gene. (C) The validation of DAS events in SOL and EDL. The first lane to fourth lane is PDE4DIP gene. The fifth lane to eighth lane is NEB gene. The ninth lane to twelfth lane is ZNF280D gene. (D) The validation of DAS events in SOL and EDL. The first lane to fourth lane is PDE4DIP gene.
